# Physical Activity and Adolescent Mental Health in China: The Mediating Role of Social Capital

**DOI:** 10.3390/bs16071102

**Published:** 2026-07-02

**Authors:** Jiankun Liu, Yueyun Zhang

**Affiliations:** 1 School of Humanities and Social Sciences, Harbin Engineering University, Harbin 150001, China; jkliu@hrbeu.edu.cn; 2 School of Philosophy and Social Development, Shandong University, Jinan 250100, China

**Keywords:** physical activity, adolescent mental health, social capital, Chinese adolescents

## Abstract

Background: Adolescent mental health is critical for healthy development, yet mental health issues remain prevalent globally. Physical activity (PA) has been linked to mental health benefits, but evidence among adolescents is inconsistent, and mechanisms remain underexplored. Methods: We analyzed cross-sectional observational data drawn from the 2014 wave of the China Education Panel Survey (CEPS), which surveyed 9920 students in total. After excluding cases with missing values on any focal variable, 7995 Chinese middle school students were retained for analysis to examine the association between PA and mental health and, in particular, the mediating role of social capital (as captured by social networks, adherence to collective norms, and trust). We employed regression and mediation analyses. Results: We found that greater PA was positively associated with better mental health, operationalized as reduced affective–symptomatic symptoms. This relationship was mediated by two dimensions of social capital—social networks and adherence to collective norms—while trust did not serve as a significant mediator. Conclusions: These findings suggest that PA may enhance adolescent mental health by fostering social connections and group engagement. Promoting PA and supportive social environments in school settings could contribute to improved mental well-being among adolescents.

## 1. Introduction

Adolescent mental health is crucial for healthy development during this formative stage of life. A recent Lancet Commission evaluation of 70 years of Chinese child and adolescent health indicates that, despite significant improvements in overall health outcomes from infancy to adulthood in recent decades, adolescent mental illness remains a major challenge in China (Qiao et al., 2021). The 2018 China Youth Development Report estimates that approximately 30 million children and adolescents under 17 in China experience various mental health problems (National Health Commission of the People’s Republic of China, 2018). This high prevalence has raised significant concern among educators and policymakers. In response, the Healthy China Action (2019–2030), released by the National Health Commission in 2019, mandates the implementation of a “Mental Health Promotion Action” to reduce the increasing prevalence of mental disorders by 2022 and 2030 (National Health Commission of the People’s Republic of China, 2019). Therefore, effective interventions are urgently needed to promote adolescent well-being and strengthen national human capital.

Physical activity (PA) is widely recognized as an effective approach to mitigating mental health issues, as supported by empirical studies (Rosenbaum et al., 2014; Singh et al., 2023). While some research has demonstrated an anti-depressive effect of PA across various age groups, including children and youth, in cross-national data (Schuch et al., 2018), and a recent overview of 79 systematic reviews confirmed the benefits of PA for alleviating symptoms of depression, anxiety, and distress in adults (Singh et al., 2023), findings among adolescents have been inconsistent. Some longitudinal studies report that PA does not reduce depression among adolescents and may even be associated with increased anxiety in certain subgroups (Toseeb et al., 2014; Buchan et al., 2021). Therefore, further empirical research is needed to clarify the effect of PA on adolescent mental health (Hou et al., 2025).

Moreover, most existing studies on the relationship between PA and mental health have tended to focus on college students (Silber & Garn, 2023; Wiedenman et al., 2023; Zhu et al., 2023) or have relied on small-scale surveys of teenagers (Y. Liu et al., 2024; Zhang et al., 2023). Consequently, it is unclear whether these findings are generalizable to the broader population of Chinese adolescents. Furthermore, while existing research has proposed several mechanisms linking PA to mental health, these explanations have predominantly centered on physiological and psychological pathways, leaving the role of social mechanisms comparatively underexplored. In particular, social capital, which encompasses the peer networks, collective norms, and trust relations embedded in adolescents’ social lives, provides a theoretically compelling but empirically underdeveloped account of how PA may benefit mental health in this age group. To address these gaps, the present study utilizes nationally representative sample data to investigate whether and how physical activity can be linked to adolescent mental health in the Chinese context, with a specific focus on the mediating role of multidimensional social capital.

## 2. Literature Review

### 2.1. Physical Activity (PA) and Mental Health

The health production function, initially suggested by Grossman (1972), posits that although an individual’s self-reported health status eventually declines with age, investment in personal health aids in preserving physical fitness. The elements influencing health investment include physical characteristics, socioeconomic status, natural environment, and social environment. Additional research indicates that this theory can be utilized to examine aspects associated with mental health (Fleitlich & Goodman, 2001).

Physical activity (PA) is a common form of health investment, with its most immediate benefit being the improvement of physical fitness (Hallal et al., 2006; Joensuu et al., 2024). Beyond physical benefits, numerous studies have demonstrated that PA is linked to elevated mental health. For example, Mammen and Faulkner (2013) found that PA was associated with greater relief from depression than traditional medication or psychotherapy. Daley and Jolly (2012) reported that exercise therapy was associated with reductions in depressive symptoms in individuals with both moderate and severe depression. Furthermore, Schuch et al. (2018), analyzing data from countries across Asia, Europe, North America, and Oceania, observed that exercise therapy was associated with significantly lower prevalence of depression among both children and adults.

Studies on Chinese residents have also found that PA has a positive relationship with mental health. M. Li et al. (2015) investigated 1601 college students and found that PA had a strong positive effect on student’s mental health. F. Liu et al. (2021) compared the effect of PA on mental health between college students and middle school students and found that PA had a greater positive effect on mental health among middle school students than among college students. Ren and Li (2020) found that exercise intervention is significantly related to improved mental health among left-behind children through perceived support in China.

Adolescence is a critical period for socialization. However, overwhelming academic stress—a common issue in many Asian countries—can negatively affect adolescents’ development (Shek et al., 2021). Participating in group sports provides opportunities for students to relieve academic and psychological pressure (Patterson et al., 2023). Furthermore, engaging in physical activity allows teenagers to fulfill their need for social belonging through interactions with peers, thereby reducing psychological distance and improving mental health (Watt & Kehoe, 2022).

### 2.2. The Mediating Role of Social Capital

A key question remains: what mechanisms underlie the association of PA with teenagers’ mental health? Most previous studies have examined the association between PA and adolescent mental health without thoroughly exploring the mechanisms involved. This lack of understanding can hinder the development and implementation of effective policies. While some researchers have proposed explanations, these have primarily focused on physiological and psychological factors, such as physical function (Zhu et al., 2023), self-perception of health (Gu et al., 2021; Joensuu et al., 2024), and self-efficacy (Medrano-Ureña et al., 2020; Wiedenman et al., 2023). In contrast, the present study focuses on the mediating role of social factors, specifically social capital. Adolescence is a developmental stage during which social relationships are particularly salient and formative; for this reason, social capital, which can be shaped substantially through peer interactions in school settings, may be especially responsive to PA participation and especially consequential for mental well-being.

Both individual mental health and PA are closely related to social interaction. Individuals are embedded in social networks through their interactions, and their position and resources within these networks can significantly affect their mental state. For example, studies have shown that interpersonal communication has a strong positive effect on happiness, surpassing the influence of demographic and socioeconomic factors (Su et al., 2017). Additionally, many forms of PA are highly interactive, enabling individuals to build and strengthen interpersonal connections, which can foster a more positive mindset (Patterson et al., 2023; Watt & Kehoe, 2022). For instance, Bosscher (1993) found that group exercise had a substantial effect on individual happiness, whereas solitary exercise did not. Ryff and Keyes (1995) suggested that PA can provide both material and emotional support through social connections, thereby improving well-being. Given that social interaction is a key component of social capital, this study examines whether social capital mediates the relationship between PA and mental health among Chinese middle school students.

Social capital is a concept originating in sociology that has also attracted considerable attention from researchers in economics and political science. Broadly, social capital refers to the resources accumulated through interpersonal relationships (Coleman, 1988; Y. Li & Song, 2025). Although definitions of social capital vary across disciplines (Adler & Kwon, 2002), it is widely recognized that its core element is “connection”—that is, social capital depends on the social networks formed by interpersonal interactions, social trust, and shared social norms. The primary function of social capital is to provide resources and norms for individuals within a network. These resources can be material or non-material, both of which have been shown to benefit mental health. For example, financial support from social networks can alleviate financial stress, while emotional support and a sense of security from network members (Haines & Hurlbert, 1992; Y. Li & Song, 2025), as well as the establishment of collective identity and a sense of belonging and honor (Crossley & Langdridge, 2005), contribute to psychological well-being.

Social networks, social norms, and interpersonal trust are the main forms of social capital (Bhandari & Yasunobu, 2009), and each of these dimensions can contribute to individuals’ subjective psychological well-being. First, social networks are characterized by their size—the number of people with whom an individual interacts—and their complexity, which refers to the diversity of these contacts. Individuals who interact with people of higher social status may gain access to more social resources. Thus, larger and more diverse social networks are likely to provide greater resources. Second, participation in collective activities reflects individuals’ compliance with social norms, which underpins ongoing social interactions. Through shared activities, groups develop common lifestyles and values, fostering a sense of belonging that can help relieve negative emotions and enhance resilience. For example, Putnam (2000) observed that declining participation in informal groups in the United States after World War II was accompanied by a continuous decline in happiness. Third, interpersonal trust refers to the positive expectation that others will cooperate under uncertain conditions, reflecting the closeness of relationships within a network (Fafchamps, 2003; B. Li et al., 2025). Trusting others implies confidence that they will not act opportunistically and will provide support in times of need, which can help individuals feel safe, relaxed, and positive (Bartolini et al., 2017).

PA serves as an important avenue for social interaction, enabling individuals to acquire social capital through participation. For adolescents, PA is often motivated by recreation, fitness, and personal interest rather than competition. Although many people begin exercising to improve physical fitness, the process—particularly in team sports—also facilitates interpersonal interactions. These interactions help foster social networks, encourage compliance with group norms, and build interpersonal trust, all of which are foundational elements of social capital. To successfully complete exercise tasks, participants must share experiences and collaborate, which helps them get to know each other and form long-term relationships. Participation in physical activity promotes cooperation and a sense of belonging to the team, strengthening emotional connections among members. Moreover, cooperation in sports requires trust and adherence to group norms. Thus, beyond providing a platform for socialization, PA helps individuals build close ties and trust. The positive effects of PA on various forms of social capital have been supported by numerous empirical studies (B. Li et al., 2025; Patterson et al., 2023; Rodgers et al., 2019; Zhou & Guo, 2023).

In summary, numerous studies have demonstrated that PA significantly enhances both social capital and mental health. Based on this evidence, we hypothesize that PA is positively linked to mental health among Chinese adolescents, in part by enhancing social capital through fostering interpersonal trust and norm compliance. For adolescents, engaging in physical activity facilitates interactions with classmates and serves as an important means of acquiring social capital. Accordingly, this study tests the following two hypotheses:

**H1.**  *PA (indexed by sport time) is positively associated with adolescent mental health.*

**H2.**  *Three forms of social capital (network, norm, and trust) mediate the association of PA with adolescent mental health.*

## 3. Materials and Methods

### 3.1. Data and Participants

To test the relationship between PA and adolescent mental health, the study analyzed the dataset of the China Education Panel Survey (CEPS, 2014–2015). The CEPS, launched by National Survey Research Center (NSRC), is a longitudinal nationwide survey of middle school students, parents and educators in China. At the time of data collection, the survey was designed to reflect the status of China’s middle school education. This survey adopts various sampling methods including multilevel, multistage, and probability proportional to size sampling (PPS). The 2013 baseline survey covered 19,487 middle school students in 438 classes at 112 middle schools across China. The 2014 survey tracked 9499 seventh graders from the baseline study (eighth graders at the time of the 2014 survey) and additionally surveyed 471 eighth graders (9920 students in total). Due to the availability of focal variables, this study selects the 2014 survey data for empirical analysis, thereby using a cross-sectional design. Participants were eligible for inclusion if they were enrolled students present at the 2014 survey wave with valid responses on all focal variables. Cases with missing values on any focal variable were excluded listwise (n = 1925; 19.4% of the original sample), yielding a final analytic sample of 7995 students. To assess potential selection bias introduced by listwise deletion, we compared the excluded cases with the retained sample on key demographic variables; no substantively meaningful differences were observed.

### 3.2. Measures

**Mental health.** CEPS (2014–2015) adopts a mental health scale consisting of 10 questions on teenagers’ mental health (i.e., “have you experienced any of the following feelings in the past 7 days?”), including: (1) depressive; (2) too depressive to concentrate; (3) unhappy; (4) life is boring; (5) cannot get motivated to work; (6) sad; (7) nervous; (8) worry too much; (9) feeling something bad is going to happen; (10) energetic and not attentive in class (Zhang et al., 2021a). The answer ranges from never (1) to always (5). We convert the scale into a range from 0 to 1—always (0), often (0.25), sometimes (0.5), often (0.75) and never (1)—and take the mean of the 10 indicators of the respondents and multiply them by 100 to obtain a continuous variable (m_health) with a range from 0 to 100. The higher the score, the better the respondent’s mental health. Second, we carry out principal component factor analysis on 10 mental health indicators, and rotate by the maximal varimax method. The factor loading is 3.204, the KMO value is 0.929, and the Cronbach’s α is 0.913. According to the principle that the eigenvalue is greater than 1, we extract one factor from the 10 indicators and name it as “m_health_factor”. The larger the value of mental health factor (m_health_factor), the better the mental health. It should be noted that this scale captures the affective–symptomatic dimension of mental health; higher scores reflect reduced negative emotional states rather than a comprehensive assessment of psychological well-being or functioning.

**Physical activity (PA).** The measure of PA in CEPS (2014–2015) consists of two questions: the amount of days per week for PA, and the amount of minutes per day for PA (Chen & Yao, 2025). After eliminating implausible outliers (values exceeding 360 min per day; n = 32, 0.32% of the original sample, likely reflecting response error rather than genuine high-activity profiles), we calculate the average daily exercise time (sport_time) in minutes. In addition, because students’ PA time is influenced by the campus environment, intra-school comparisons are more meaningful than cross-school comparisons. To address this problem, the sport_dummy variable was created. Specifically, students whose sport time is longer than the average sport_time of the sample schools were coded as active (1), and students whose sport_time is lower than the average were coded as inactive (0).

**Potential mediators.** This study hypothesizes that social capital is the mediator by which PA improves adolescent mental health. In previous empirical studies, social capital is usually operationalized as three indicators: network, norm and trust (Bhandari & Yasunobu, 2009). Accordingly, we used three social capital indicators as follows:

(1) Network. The closeness with classmates is used to measure network. The questions in the questionnaire are “most of my classmates are friendly to me” and “I feel close to my classmates”. The respondents reported how they agree with the statements from totally disagree (0) to completely agree (3). The network variable was measured by averaging the scores of these two questions. The larger the score, the stronger the student network.
(2) Norm. Two items of CEPS (2014–2015) are selected to assess students’ compliance with collective norms: “I often participate in activities organized by my school or my class?” and “my class has a good atmosphere”. The answer ranges from totally disagree (0) to completely agree (3). The norm variable was measured by averaging the scores of the two items. The larger the score, the more the respondents complied with collective norms.
(3) Trust. Trust was measured by the item “who do you first turn to whenever you want somebody to talk with” in the questionnaire. Choosing classmates was dummy coded as 1 (high trust in classmates). Choosing parents, relatives, school teachers, or no one to find was dummy coded as 0 (low trust in their classmates).

**Covariates.** To minimize potential confounding, we controlled for a series of covariates. Individual, family, and school-level exogenous variables were controlled in this study (Chen & Yao, 2025; Guan & Tena, 2021; Xia et al., 2020). Individual variables included student’s age (st_age), student’s gender (st_gender: female = 0, male = 1), whether he/or she was the only child in the family (one_child: no = 0, yes = 1) and the household region (st_hukou: rural = 0, non_rural = 1). Family level variables were parental education (p_edu), parental occupational status (p_occu), family socioeconomic status (family_SES), and parent–child relationship (relationship). In this study, the highest educational level of parents is operationalized as the educational year, which was coded according to the stages of education: no education (0 year), primary school (6 years), middle school (9 years), technical school/ high school (12 years), vocational college (15 years), undergraduate (16 years), and postgraduate or above (19 years). In the existing literature, managers and professional personnel are usually regarded as groups with high occupational status (Song et al., 2020), and the corresponding occupational categories in CEPS are as follows: (1) government officials; (2) enterprise management; (3) university teachers, scientists, engineers and other professional and technical personnel; (4) doctors, lawyers and primary and secondary school teachers. Therefore, if the parents are engaged in any of the above occupations, they were regarded as people having high occupational status (coded as 1); otherwise, parents engaged in other occupations were regarded as the low occupation group (0). Family socioeconomic status (family_SES) are measured by student’s assessment of their current family financial status, ranging from very difficult (coded as 1) to very wealthy (5). Parent–child relationship was measured through student’s evaluation of their closeness to their father and mother, respectively, from not close (0), medium (1) to very close (2). We combined the two indicators and average them to construct a continuous variable of parent–child relationship. School-level variables were school educational quality (sch_rank), school sports facilities (facility), and the regional characteristics of school (sch_urban and sch_district). School educational quality refers to the local ranking of the school ranging from worst (0), worse (1), middle (2), better (3), and best (4). School sports facilities include playing fields, gym and swimming facilities. CEPS (2014–2015) asked the principals of each school about the completeness of the three types of sports facilities, and the answer options are none (0), have facilities but needs to be improved (1), or have well equipped facilities (2). In this study, the sum of the scores of the three types of sports facilities constituted a continuous variable (facility). The sch_urban (urban = 1, rural = 0) and sch_district (east = 0, central = 1, west = 2) are the locations of schools.

### 3.3. Analytical Strategy

The data analysis in this paper comprises descriptive statistics and regression analyses. Descriptive statistics summarize all variables and include mean difference tests. The regression analyses investigate the impact of PA on adolescent mental health and assess whether social capital serves as a mediator, including both baseline regressions and mediation effect tests.

A multiple linear regression model is used to estimate the direct effect of PA on mental health.

(1) $${m\_health}_{i}=\alpha_{0}+\beta_{1}\times {sport}_{i}+\beta_{2}{\times X}_{i}+\varepsilon_{i}$$

In Formula (1), ${m\_health}_{i}$ represents student *i*’s mental health score, and ${sport}_{i}$ represents the average sport time of student *i* each day. ${Χ}_{i}$ represents a group of control variables. $\varepsilon_{i}$ is a random perturbation term. In this formula, we care most about *β*_1_. If *β*_1_ > 0, it means that PA has a positive effect on mental health.

Then, we adopt the three step method to test the mediating effects as follows:

(2) $${m\_health}_{i}=\alpha_{0}+\beta_{1}\times {sport}_{i}+\beta_{2}{\times X}_{i}+\varepsilon_{i}$$

(3) $${SC}_{i}{=\alpha }_{1}+\gamma_{1}\times {sport}_{i}+\gamma_{2}\times X_{i}+\varepsilon_{i}$$

(4) $${m\_health}_{i}=\alpha_{0}^{\prime }+\beta_{1}^{\prime }\times {sport}_{i}+\gamma_{1}^{\prime }\times {SC}_{it}{+\beta }_{2}^{\prime }\times X_{i}+\varepsilon_{i}^{\prime }$$

Equation (3) is the regression from student i’s social capital to sport time. Equation (4) is the equation for the mediating effect of social capital between students’ PA and mental health. Three mediators (network, norms, and trust) were included in the model, with mental health as the dependent variable and student’s sport time as the independent variable, and the change in the independent variable coefficient $\beta_{1}^{\prime }$ was tested. If the coefficient of $\gamma_{1}^{\prime }$ is positive and statistically significant, and the significance of $\beta_{1}^{\prime }$ is reduced or the coefficient value is reduced, we can conclude that PA can predict adolescent mental health through social capital.

Because we used three indicators of students’ social capital, KHB method (Karlson et al., 2012) is adopted to calculate the specific contribution rate of each indicator of social capital to the overall mediating effect. KHB method is similar to stepwise regression, while it enables us to directly compare the effects of each mediation variables.

## 4. Results

### 4.1. Descriptive Results

Table 1 presents the sample description. The mean self-rated mental health score among adolescents is 71.03, and the mental health factor score is 0.02. The average daily sport time is 20.61 min, with 41% of teenagers exercising regularly. Regarding mediating variables, the mean network score is 2.13, suggesting close relationships and smooth communication with classmates. The mean norm score is 1.98, indicating relatively high participation in collective activities and generally positive class atmospheres. The mean trust score is 0.74, meaning that 74% of teenagers chose classmates as their first choice for social support, reflecting a high level of trust among classmates. The average age is 13.91 years, with males comprising 51% of the sample and 45% being only children. About 47% have non-rural household registration (st_hukou). The average parental education is 12.57 years, and 11% of parents are managers or professionals. A total of 73.52% of adolescents rated their family SES as middle class. Most sample schools are ranked above average and have at least one sports facility. Additionally, 83% of schools are in urban areas, 55.01% are in eastern China, and 24.84% are in the west.

Differences in dependent and mediating variables between active and inactive student groups were tested, with results reported in Table 2. The active group (Group-1) scored significantly higher than the inactive group (Group-0) in both mental health and the mental health factor (*p* < 0.001). This suggests a preliminary positive association between PA and adolescent mental health, which is further examined in the regression analyses. Additionally, the inactive group scored significantly lower on all three indicators of social capital compared to the active group (*p* < 0.05), indicating that teenagers who exercise regularly possess greater social capital at school.

### 4.2. Regression Results

Table 3 reports the results of multiple regression models used to estimate the impact of PA on adolescent mental health. The dependent variable of Model 1 and Model 3 was adolescents’ self-rated mental health, and the dependent variable of Model 2 and Model 4 was the mental health factor. The independent variable of Model 1 and Model 2 was PA time. The results showed that the coefficient of sport_time was 0.029 and 0.001, respectively (*p* < 0.05). The independent variable of Model 3 and Model 4 was dummy-coded PA variable. The mental health score of the active adolescents was 1.430 points higher than that of the inactive adolescents (*p* < 0.001), and the mental health factor score of the active adolescents was 0.062 points higher than that of inactive adolescents (*p* < 0.001). The results indicate that adolescents’ mental health level is positively associated with PA levels, providing support for Hypothesis 1.

On the basis of the relationship between PA and adolescent mental health, a three-step method was adopted to examine the mediating effects of three social capital indicators (network, norm, and trust), with adolescents’ self-rating mental health (m_health) as the dependent variable. Results are shown in Table 4. In the first step, the dependent variable (m_health) was regressed on the independent variable (sport_time; Model 1). In the second step, norm, trust, and network were separately regressed on the independent variable in Models 2, 4, and 6. In the third step, the dependent variable (m_health), mediating variables, and independent variable (sport_time) were all included in Models 3, 5, and 7, and the resulting coefficients were compared with those in the first step to determine mediation.

**Figure 1:**
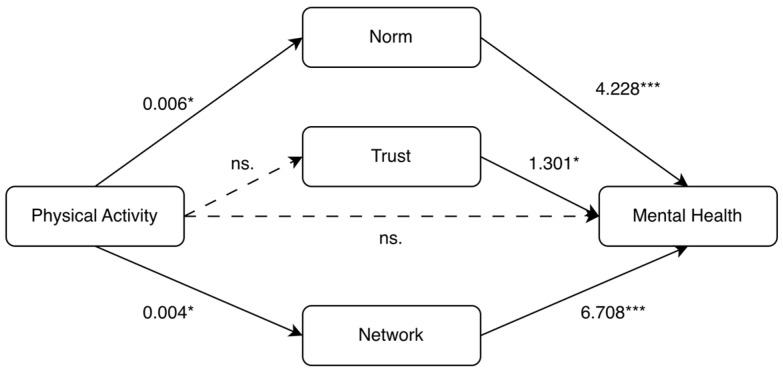
Path diagram illustrating path coefficients obtained from models in Table 4. * *p* < 0.05, *** *p* < 0.001; ns denotes non-significant.

Results from Models 2, 4, and 6 showed that sport_time was positively associated with norm and network, but showed no significant association with trust. In Models 3 and 7, norm and network were each significantly associated with adolescents’ mental health. Compared with Model 1, the coefficients of sport_time in Model 3 and Model 7 became non-significant (β = 0.005, SE = 0.014; β = 0.003, SE = 0.014), suggesting that the associations between physical activity and mental health were fully accounted for by norm and network. These results are consistent with full mediation through school-based peer relational resources, while the indirect pathway through trust was not supported. Figure 1 illustrates the corresponding path diagram.

To further decompose and compare the relative magnitude of the two indirect pathways, the KHB method was adopted. The KHB decomposition partitions the total effect of sport_time (β = 0.030) into a direct effect (β = 0.002) and a total indirect effect (β = 0.028) operating through the two social capital mediators, with the indirect component accounting for 93.33% of the total effect. The results in Table 5 showed that both indirect effects were statistically significant. Among the two pathways, the indirect effect through network (β = 0.026, 86.81% of total indirect effect) was substantially larger than that through norm (β = 0.024, 13.19% of total indirect effect), suggesting that peer network integration plays a more prominent role in linking physical activity to adolescent mental health than normative conformity.

## 5. Discussion

Mental health plays an important role in the development of adolescents. Studies have shown that mental problems have become a significant public health problem (Patton et al., 2016), and adolescents have become a high-risk group for suffering from mental problems (Irwin, 2017; Orben et al., 2024; Zhang et al., 2021b). By analyzing data from CEPS (2014–2015), this study found that PA was significantly associated with better adolescent mental health (as indexed by reduced affective symptomatic distress), consistent with the findings of Schuch et al. (2018) and Guan and Tena (2021). Although researchers in China have expressed concern about adolescent mental health problems, previous studies have not been based on nationally representative samples.

This study addresses this gap by using nationally representative data and demonstrates that PA is an important factor associated with adolescent mental health, indicating that PA is linked to greater subjective well-being among teenagers. While the coefficient for sport_time is modest in magnitude (β = 0.029), this should be interpreted alongside the finding that adolescents classified as active scored 1.430 points higher on the mental health scale than their inactive peers (*p* < 0.001). Furthermore, given the large, nationally representative sample, even modest individual-level associations may carry meaningful public health significance at the population level. Notably, the primary contribution of this study lies not in the overall effect size of PA, but in elucidating the social mechanisms through which PA operates—an issue addressed in the following discussion.

More importantly, this study explored the mechanisms linking PA to adolescent mental health through the lens of social capital, finding that peer network integration and normative conformity—but not interpersonal trust—fully mediated this association. These differential patterns warrant substantive explanation.

Physical activity in school settings is inherently social: it expands peer contacts, creates opportunities for cooperation, and embeds adolescents in shared group structures. Through these processes, PA is associated with richer peer networks, which in turn provide emotional support, informational resources, and a sense of belonging—all well-documented protective factors for mental health (Haines & Hurlbert, 1992; Rodgers et al., 2019). The mediation through normative conformity reflects a related but distinct pathway: participation in collective physical activities reinforces shared behavioral standards and group identity, which may reduce feelings of alienation and provide adolescents with a stable social reference frame conducive to psychological well-being (Ren & Li, 2020).

The absence of a significant indirect effect through trust, however, is theoretically instructive. Trust—particularly the generalized interpersonal trust measured in this study—is not a resource readily activated through short-term behavioral participation. Research on trust development suggests that trust among adolescents is built gradually through repeated, reciprocal interaction over time, rather than being a proximate outcome of shared activity (Rotenberg, 2010). It is also worth noting that the trust item used here captures a preference for confiding in peers, which may reflect pre-existing relationship quality rather than social capital generated by PA. Taken together, these findings suggest that network-level integration and normative alignment are the more proximal social mechanisms through which PA may be linked to mental health benefits.

These findings carry several practical implications for school-based interventions. First, given that social networks and collective norms—rather than generalized trust—emerged as active mediating pathways, schools should consider the social context of physical activity, not merely its frequency or duration. While the present study did not directly measure exercise type, the mediation findings suggest that group-based forms of PA may be particularly beneficial, as they are more likely to foster peer interaction and normative engagement. Future research directly comparing team-based and individual exercise would help confirm this interpretation. Second, schools should pay particular attention to students who are less socially integrated—such as new entrants or students on the social periphery—since these students are least likely to benefit from the network and norm pathways identified here. Targeted PA programs designed to facilitate peer inclusion for such students could maximize the mental health returns of physical education policy. Third, the non-significant mediation of trust suggests that PA participation, at least at the intensity and duration typical in middle school settings, does not reliably cultivate generalized interpersonal trust. Interventions aimed specifically at building trust among adolescents may require more sustained, relationship-focused approaches beyond routine physical education.

This study has several limitations that should be acknowledged. First, the analyses rely on the CEPS 2014–2015 wave, as more recent large-scale survey data with equivalent measures are not currently available. Given the substantial changes in Chinese adolescent life since then—particularly the proliferation of digital media and the COVID-19 pandemic—the generalizability of these findings to the current context remains uncertain. Second, the cross-sectional design of the CEPS precludes causal inference, and reverse causality cannot be ruled out: adolescents with better mental health may be more inclined to engage in physical activity and to build social connections. In particular, the mediation analyses should be interpreted with caution: although we follow established procedures (regression steps and KHB decomposition), the temporal precedence of physical activity over social capital and mental health cannot be confirmed from cross-sectional data, and the mediation pathways are better understood as conditional associations than causal mechanisms. Third, the operationalization of physical activity is relatively coarse, capturing only frequency and duration. Information on exercise intensity, type (e.g., team-based vs. individual), and the voluntary or compulsory nature of participation was unavailable—a notable constraint given that our proposed mechanism relies substantially on social interaction as a mediating pathway. Fourth, the social capital measures represent school-based proxies derived from available CEPS items rather than validated scales. As this study draws on secondary data from the CEPS—a large nationally representative survey—the selection of items was determined by the original survey design rather than by the theoretical requirements of the present study, precluding the use of purpose-built instruments such as validated adolescent social capital scales. This limits construct validity. This concern is most acute for trust, which is operationalized with a single item—whether students first turn to classmates when they want to talk—and may capture help-seeking preference rather than generalized interpersonal trust. This measurement constraint may also partially account for the null mediation finding for trust. Taken together, these measurement constraints suggest that the mediation findings are best understood as associations among operationalized proxies rather than as tests of the theoretical constructs in their broader conceptual sense. Finally, the sample is restricted to middle school students within the CEPS sampling frame, limiting generalizability to other age groups.

Future research should pursue several specific directions. First, replicating the mediation model with post-2020 data would allow researchers to assess whether the network and norm pathways remain operative in a context shaped by the COVID-19 pandemic and intensified digital media use. Second, the social capital measures in the present study are school-based proxies derived from available survey items; future work should employ validated multi-item scales—such as those measuring perceived social support, peer network density, and norm internalization separately—to more precisely capture each dimension and reduce measurement error. Third, longitudinal or experimental designs, such as randomized PA interventions with follow-up assessments, would help establish the causal direction of the relationships identified here and rule out the reverse causality concern noted above.

## Figures and Tables

**Table 1:** Descriptive statistics.

Variables	obs	Mean	Variable	Observations	Percentage
m_health	7995	71.03	family_SES		
m_health_factor	7995	0.02	very difficult	212	2.65
sport_time	7995	20.61	difficult	981	12.27
sport_dummy	7995	0.41	middle	5878	73.52
network	7995	2.13	wealthy	869	10.87
norm	7995	1.98	very wealthy	55	0.69
trust	7995	0.74	sch_rank		
st_age	7995	13.91	worst	30	0.38
st_gender	7995	0.51	worse	530	6.63
one_child	7995	0.45	middle	1026	12.83
st_hukou	7995	0.47	better	4231	52.92
p_edu	7995	12.57	best	2178	27.24
p_occu	7995	0.11	sch_district		
relationship	7995	1.61	east	4398	55.01
facility	7995	1.24	central	1611	20.15
sch_urban	7995	0.83	west	1986	24.84

**Table 2:** Two-sample mean tests.

Variables	Group-0	Mean-0	Group-1	Mean-1	Difference
m_health	4754	70.160	3241	72.311	−2.151 ***
m_health_factor	4754	0.017	3241	0.077	−0.060 ***
network	4754	2.070	3241	2.212	−0.142 ***
norm	4754	1.904	3241	2.098	−0.194 ***
trust	4754	0.733	3241	0.753	−0.020 **

Note. ** *p* < 0.01, *** *p* < 0.001.; Group-0 (inactive group) is the students whose exercise time is shorter than the average of school, and Group-1 (active group) is the students whose exercise time is longer than the average of school.

**Table 3:** Regression of mental health on physical activity (PA).

Variables	Model-1	Model-2	Model-3	Model-4
	m_health	m_health_factor	m_health	m_health_factor
sport_time	0.029 **	0.001 **		
	(0.014)	(0.001)		
sport_dummy			1.430 ***	0.062 ***
			(0.497)	(0.022)
st_age	−1.143 ***	−0.049 ***	−1.115 ***	−0.048 ***
	(0.300)	(0.013)	(0.300)	(0.013)
st_gender	2.144 ***	0.096 ***	2.163 ***	0.097 ***
	(0.495)	(0.022)	(0.491)	(0.021)
one_child	0.627	0.028	0.650	0.029
	(0.561)	(0.024)	(0.561)	(0.024)
st_hukou	0.745	0.032	0.821	0.036
	(0.560)	(0.024)	(0.560)	(0.024)
p_edu	0.375 ***	0.016 ***	0.373 ***	0.016 ***
	(0.077)	(0.003)	(0.077)	(0.003)
p_occu	1.942 **	0.084 **	1.947 **	0.084 **
	(0.972)	(0.042)	(0.971)	(0.042)
family_SES				
difficult	2.098	0.090	2.092	0.090
	(1.958)	(0.085)	(1.957)	(0.085)
middle	6.952 ***	0.302 ***	6.995 ***	0.303 ***
	(1.878)	(0.082)	(1.877)	(0.082)
wealthy	8.439 ***	0.367 ***	8.511 ***	0.370 ***
	(2.036)	(0.089)	(2.034)	(0.089)
very wealthy	6.462	0.283	6.549	0.287
	(4.398)	(0.191)	(4.409)	(0.192)
relationship	11.784 ***	0.512 ***	11.785 ***	0.512 ***
	(0.593)	(0.026)	(0.593)	(0.026)
sch_rank				
worse	0.255	0.013	−0.297	−0.011
	(5.361)	(0.234)	(5.323)	(0.232)
middle	1.935	0.086	1.474	0.066
	(5.329)	(0.232)	(5.290)	(0.231)
better	−0.114	−0.004	−0.530	−0.022
	(5.298)	(0.231)	(5.259)	(0.229)
best	−0.442	−0.019	−0.835	−0.037
	(5.319)	(0.232)	(5.281)	(0.230)
sch_facility	−0.685	−0.029	−0.665	−0.029
	(0.520)	(0.023)	(0.517)	(0.023)
sch_urban	−1.162 *	−0.050 *	−1.131 *	−0.049
	(0.684)	(0.030)	(0.685)	(0.030)
sch_district				
central	−3.109 ***	−0.134 ***	−3.268 ***	−0.141 ***
	(0.702)	(0.031)	(0.699)	(0.030)
west	−2.338 ***	−0.104 ***	−2.358 ***	−0.105 ***
	(0.639)	(0.028)	(0.639)	(0.028)
constant	57.331 ***	−0.581 *	57.281 ***	−0.583 *
	(7.372)	(0.321)	(7.349)	(0.320)
*N*	7995	7995	7995	7995
*R* ^2^	0.085	0.085	0.086	0.085

Note: * *p* < 0.05, ** *p* < 0.01, *** *p* < 0.001; robust standard errors are in the parentheses. The control variables below are the same as here.

**Table 4:** Mediation analysis.

Variables	Model-1	Model-2	Model-3	Model-4	Model-5	Model-6	Model-7
	m_health	Norm	m_health	Trust	m_health	Network	m_health
sport_time	0.029 **	0.006 ***	0.005	0.000	0.029 **	0.004 ***	0.003
	(0.014)	(0.000)	(0.014)	(0.000)	(0.014)	(0.000)	(0.014)
norm			4.228 ***				
			(0.369)				
trust					1.301 *		
					(0.762)		
network							6.708 ***
							(0.407)
constant	58.931 ***	1.576 ***	52.268 ***	0.967 ***	60.190 ***	1.593 ***	48.242 ***
	(5.171)	(0.174)	(5.103)	(0.081)	(5.265)	(0.161)	(5.023)
*N*	7995	7995	7995	7995	7995	7995	7995
*R* ^2^	0.085	0.104	0.103	0.023	0.085	0.104	0.125

Note: Figures in parentheses are standard errors. * *p* < 0.05, ** *p* < 0.01, *** *p* < 0.001.

**Table 5:** KHB decomposition.

Total Effect	Direct Effect	Mediating Effect
0.030 **	0.002	0.028 ***
(0.012)	(0.045)	(0.003)
Z-variable	Norm	Network
Indirect effect	0.024 ***	0.026 ***
	(0.000)	(0.000)
Percent	13.19	86.81

Note. The KHB method decomposes the total effect of sport_time into a direct effect (the coefficient net of mediators) and a total mediating effect (the difference between total and direct effects). “Percent” refers to each mediator’s share of the total indirect effect, not of the total effect. Standard errors in parentheses. ** *p* < 0.01, *** *p* < 0.001.

## Data Availability

The data that support the findings of this study are available from the CEPS project site, subject to registration and application process. Further details can be found at http://ceps.ruc.edu.cn/ (accessed on 25 January 2024). We agreed to share the data from this study conditional upon permission from the CEPS project.
